# Guarding against the most dangerous emerging pathogens.

**DOI:** 10.3201/eid0204.960401

**Published:** 1996

**Authors:** P W Ewald

**Affiliations:** Department of Biology, Amherst College, Amherst, MA 01002-5000, USA. pwewald@amherst.edu

## Abstract

Control of emerging infectious diseases will be difficult because of the large number of disease-causing organisms that are emerging or could emerge and the great diversity of geographic areas in which emergence can occur. The modern view of the evolution of pathogen virulence--specifically its focus on the tradeoff between costs and benefits to the pathogen from increased host exploitation--allows control programs to identify and focus on the most dangerous pathogens (those that can be established with high virulence in human populations).


### Guarding Against the Most Dangerous Emerging Pathogens: Insights from Evolutionary Biology

**Paul W. Ewald**
Department of Biology, Amherst College,
Amherst, Massachusetts, USA

Control of emerging infectious diseases will be difficult because of the large number of disease-causing organisms that are emerging or could emerge and the great diversity of geographic areas in which emergence can occur. The modern view of the evolution of pathogen virulence—specifically its focus on the tradeoff between costs and benefits to the pathogen from increased host exploitation—allows control programs to identify and focus on the most dangerous pathogens (those that can be established with high virulence in human populations).

Studies of emerging diseases have focused chiefly on the spectrum of different emerging pathogens, epidemiologic reasons for emergence, and interventions to control emergence. The feasibility of disease control is hampered by the potentially vast number of emerging and reemerging pathogens, the diversity of geographic sources, the potential for rapid global dissemination from these sources, and numerous ecologic and social factors influencing emergence (1-4). Disease control could be made more manageable if the most dangerous pathogens could be singled out for the most intense study, surveillance, and control efforts. Experts who have addressed this problem from an epidemiologic but not an evolutionary perspective disagree about the feasibility of predicting and preventing the emergence of the most damaging new pathogens (5-8). In this perspective, I argue that improved understanding of the evolution of virulence (defined broadly as the harmfulness of an infection) can make this goal more feasible in two ways: 1) by facilitating identification and blocking of pathogens that represent the greatest threat should they become established in human populations (e.g., *Yersinia pestis* during the Middle Ages and human immunodeficiency virus [HIV] during recent decades) and 2) by providing methods for inhibiting the emergence of particularly virulent variants of pathogens that are already established in human populations (e.g., the pathogen that caused the 1918 influenza pandemic and virulent, antibiotic-resistant strains of *Staphylococcus aureus)*.

Modern understanding of the evolution of virulence focuses on a tradeoff to which pathogens are subjected: the competitive benefits that pathogens accrue through increased exploitation of hosts and the costs that result from any effects of disease that reduce infectious contact between infected and susceptible hosts. The traditional view presumed that natural selection would favor evolution toward benign coexistence between host and parasite (9-12). The modern view, however, stresses that such benign coexistence will be unstable if pathogens that exploit hosts to a greater degree have more overall success across transmission cycles than those that achieve benign coexistence (13-17).

The primary assumption of this evolutionary argument is that increased virulence is correlated with increased pathogen propagation (manifested as increases in pathogen reproduction within hosts and/or pathogen shedding from infected hosts). This correlation need not be strong across host/pathogen associations for the arguments to be valid; differences in pathogenic mechanisms, for example, could make the correlation virtually undetectable when extremely different kinds of

Address for correspondence: Paul W. Ewald, Department of Biology, Amherst College, Amherst, MA 01002-5000; fax: 413-542-7955; e-mail: pwewald@amherst.edu.

pathogens are compared. Rather, the tradeoff argument states that for a given pathogen (with its particular tropisms and pathogenic mechanisms), mutations that increase the level of host exploitation tend to increase harmfulness. The association between virulence, exploitation, and pathogen propagation is expected among "wild type" mutants, but not among novel laboratory-generated virulent ones. Because there are many routes to increased virulence and laboratory-generated variants are often not selected on the basis of competitive superiority in vivo, the increased virulence of variants generated in the laboratory may not be linked to propagative superiority. In contrast, natural selection should eliminate any variants for which increases in virulence are not linked to increases in pathogen fitness.

The connection between virulence, host exploitation, and pathogen propagation may be indirect or direct. If the pathogenic mechanism involves toxin production, a positive association is expected between toxin production and pathogen propagation. In *Vibrio cholerae*, for example, high toxin production is associated with increased densities of vibrios in the fecal material, apparently as a result of the toxin's flushing of competing organisms from the intestinal tract (17). In other organisms, the association between virulence, host exploitation, and pathogen propagation is more direct. The human plasmodia that reproduce more extensively often cause more severe illness and are more life-threatening (16). Similarly, more virulent strains of vector-borne dengue virus reproduce more extensively in cell culture (18). Growth rates of *Salmonella typhimurium* were reduced by eliminating one of its virulence plasmids and inhibiting the plasmid's expression; introduction of an 8-kb region encoding the spv genes restored increased growth rate (19). Comparison of *Shigella* species suggests a similar association between virulence and pathogen reproduction (20).

Sexually transmitted pathogens show analogous associations. For the best studied pathogen, HIV, more rapidly replicative HIVs are associated with greater cellular destruction in vitro, more rapid destruction of the immune system, and more rapid onset of AIDS (21-35). Similarly, the more oncogenic serotypes of human papilloma-viruses (HPV) generate greater numbers of progeny by interfering with the cell's mechanisms for restricting cell division (36). For both viruses, increased viral loads are associated with increased probability of transmission to contacted persons (37-39), and HIV-1, which propagates to higher densities than HIV-2, is more transmissible per contact (40).

The association between virulence and viral propagation in pathogens circulating naturally in human populations therefore supports the modern emphasis on a tradeoff between the fitness benefits and the costs accrued by pathogens as a function of changes in host exploitation.

### Transmission Associated with High Virulence

### Transmission from Immobile Hosts

Like the traditional view of host/parasite coevolution, the modern view identifies host illness as a potential liability for the pathogen. When pathogens rely on the mobility of their current host to reach susceptible hosts, the illness caused by intense exploitation typically reduces the potential for transmission. The modern perspective on host/parasite coevolution differs from the traditional one, however, in its emphasis on weighing these setbacks against the benefits of exploitation: high virulence can contribute to evolutionary stability if the costs incurred by parasites from exploitation-induced damage are particularly small and/or the benefits obtained from exploitation are particularly big. Thus, if host immobilization has little negative effect on transmission, pathogen variants that exploit the host so intensely that it is immobilized will reap the benefits of exploitation. Put more generally, when the costs incurred from transmission associated with immobilization are small, the costs of exploitation should outweigh the benefits at a higher level of exploitation—and hence virulence—than would occur if immobilization severely impaired transmission (16).

Recognizing this version of the general tradeoff led to several predictions: Because vector-borne parasites can be transmitted effectively from immobilized hosts, they should evolve to a higher level of virulence than directly transmitted parasites (16). Similarly, aspects of human behavior and culture can form “cultural vectors,” which transmit pathogens from immobile to susceptible hosts (41). For example, diarrheal pathogens that are largely waterborne should evolve to relatively high levels of virulence because effective transmission can occur even when infected hosts are mobilized: persons carrying contaminated clothing and bedding, the water used for washing bed sheets, and the movement of contaminated water into drinking water together act like a swarm of mosquitoes, transmitting pathogens from the immobilized host. Attendant-borne pathogens should also become virulent. Attendant-borne transmission often occurs in hospitals, when nurses and physicians transmit pathogens from one immobilized patient to another. A reciprocal process occurs when parasites rely on the mobility of susceptible persons rather than the mobility of the infected hosts to reach the susceptible persons. Parasites that are durable in the external environment should thus evolve toward a higher level of virulence than nondurable pathogens because durable pathogens may remain viable in the environment until the movement of susceptible individuals brings them into contact with the pathogens.

Each of these hypotheses has been evaluated and in each case the expected association occurred: virulence is positively associated with vector-borne transmission, waterborne transmission, attendant-borne transmission, and durability in the external environment (Table 1). This evolutionary framework, therefore, explains the diversity of human parasites in a way that contrasts starkly with the traditional view. Instead of being seen as a sign of maladaption, the severity of diseases such as malaria, tuberculosis, smallpox, cholera, and typhoid fever is seen as a consuequence of evolutionary adaptation because the causative parasites do not rely on host mobility for transmission. The tradeoffs between the benefits and costs of exploitation, therefore, favor evolution of relatively high levels of exploitation for such pathogens and hence high degrees of harm to the host.

### Sexual Transmission

The evolutionary tradeoffs associated with virulence in sexually transmitted diseases involve the requirements for sexual transmission imposed on the pathogens by the sexual behavior of the host. Short durations of infections would be ineffective for most sexually transmitted pathogens. If people changed sex partners once per year, for example, a pathogen that was rendered noninfectious by immunologic defenses or the host’s death within a few weeks would have little chance of being transmitted. To survive, the pathogen must be transmissible for a period that extends into the time of the next sexual partnership. To prosper, the pathogen must be transmissible for periods that span more than one change in sex partners; therefore, sexually transmitted pathogens may often need cell and tissue tropisms that keep them from being eliminated by the immune system for relatively long periods.

The evolutionary effects of changes in sexual behavior on virulence may be strongly influenced by tropisms that were present before the behavior change. Increased potential for sexual transmission should favor pathogen variants that reproduce more extensively sooner after the onset of infection. If the preexisting tropisms target nonessential cell types, this selection for earlier reproduction will have relatively little effect on virulence. If, for example, people changed sex partners every few days, the sexually transmitted pathogen should evolve virulence levels much like those of respiratory tract pathogens, which rely on host mobility for transmission. Examples of such pathogens are sexually transmitted unicellular pathogens such as *Neisseria gonorrheae* and *Chlamydia trachomatis*, which tend to infect mucosal tissues and, therefore, have relatively minor negative effects on the survival of adult hosts. If, however, the tropisms involve critical cells, the damage associated with increased levels of host exploitation should be more severe to the host. HIV provides an example: HIV has a tropism for helper T cells, which are critical regulators of immunologic responses. Although a high level of replication in these cells can be tolerated over short periods, it eventually leads (by mechanisms that are still being clarified) to the decimation of this category of cells and the collapse of the immune system.

If these arguments about evolutionary forces and tissue tropisms are applicable to

HIV, HIVs should be more virulent in areas where the potential for sexual transmission is greater. In accordance with this prediction, HIV-2 tends to be less virulent than HIV-1; moreover, evidence indicates that during the early years of HIV infection in Africa, HIV-2 tended to be transmitted in populations having a lower potential for sexual transmission (17,20). The overall validity of this approach to HIV virulence, however, will be better tested as different variants of HIV emerge in different geographic regions. Information about the potential for sexual transmission can help predict the evolution of HIV virulence in different geographic areas. On the basis of the evolutionary tradeoffs mentioned above, for example, the type E HIV-1s that are circulating in Thailand (where the potential for sexual transmission has been great) are predicted to be particularly virulent (17). Although this prediction needs to be evaluated rigorously, recently gathered data support the prediction: the decline in CD4+ cell counts of persons infected with HIV and the progression of illness in these patients appear to be particularly rapid in Thailand (43-44).

The most important application of this evolutionary approach to HIV, however, pertains to interventions that can be used to control the future evolution of HIV. If the inherent virulences of HIVs depend evolutionarily on the potential for sexual transmission, interventions that reduce this potential should have a long-term evolutionary effect, as well as widely recognized short-term epidemiologic effects—in addition to reducing the spread of HIV infection, such interventions should reduce the harmfulness per infection. Follow-up of persons infected with HIV-1 for more than a decade without deterioration of the immune system indicates that the mildness of the infections is sometimes attributable to inherently mild viruses (45-47). The raw material for this evolutionary change, therefore, appears to be already present in the HIV gene pool.

In Japan, which has a relatively low potential for sexual transmission (48), type E HIV-1s have recently been introduced from Southeast Asia. If a low potential for sexual transmission favors evolution toward mildness, the Japanese type E viruses should become milder over the next few decades.

### Assessing the Threat Posed by Pathogens

### Assessment Goals

Focusing investigative and intervention efforts on the most significant disease threats makes sense only if the threats can be reliably assessed. The long-term threat depends on the evolutionary stability of high pathogen virulence, and the most dangerous pathogens are those that threaten widespread persistence with severely damaging manifestations. One of the most important tasks in controlling emerging diseases is to identify and block such pathogens during the early stages of emergence, or better yet, before they emerge. If the most dangerous pathogens—the future analogs of the causes of AIDS, malaria, smallpox, tuberculosis, and cholera—could be effectively blocked, the effort against emerging diseases would be successful. If not, the effort may be looked on as a failure in spite of successes against pathogens that are less able to effectively penetrate human populations or relatively benign when they do establish

Table 1. Categories of pathogens that pose threats of being stably harmful in human populations because of reduced dependence on host mobility

| Characteristics allowing transmission from immobile hosts | Association with lethality | Reference |
|---|---|---|
| arthropod-borne transmission | lethality higher among arthopod-borne pathogens than among directly transmitted pathogens | (16) |
| water-borne transmission | lethality of diarrheal bacteria correlated with tendencies for waterborne transmission | (42) |
| attendant-borne transmission | lethality of *E. coli* correlated with duration of attendant-borne cycling | (20, 41) |
| durability in the external environment | lethality of respiratory-tract pathogens correlated with durability | * |

*B. A. Walther and P. W. Ewald, unpublished manuscript

Table 2. First-level checklist for identifying the most dangerous emerging pathogens. If the answer to any of the questions is yes, the potential for continuous transmission between humans should be assessed. If this potential is high, the pathogen should be considered particularly dangerous.

- Does it have a tendency for waterborne transmission?
- Is it vector-borne with the ability to use humans as part of the life cycle?
- If it is directly transmitted, is it durable in the external environment?
- Is it attendant-borne?
- Is it needle-borne?*
- If it is sexually transmitted, is it mutation-prone with a tropism for critical cell types or does it have invasive or oncogenic tendencies?

*The hypothesized importance of needleborne transmission has not yet been tested; it has been included in this listing on the basis of the harmfulness of needleborne pathogens and the hypothetical assocations between needleborne transmission and virulence (17).

themselves. The emergence, spread, and persistence of pathogens with the characteristics of rhinoviruses, for example, would not be looked on as a great failure. The establishment of such pathogens would hardly be noticed against the current backdrop of mild to moderately severe respiratory tract pathogens.

To identify pathogens that must be studied and controlled most intensively, each pathogen should be assessed for two characteristics that are associated with high virulence: 1) an ability to spread well from human to human (directly or indirectly through vectors) rather than infecting humans as dead-end hosts, and 2) transmission features that select for high levels of virulence.

The existing associations between virulence and transmission characteristics (Table 1) can be used to make such identifications. Table 2 offers a checklist that could be applied to each emerging pathogen to determine whether it makes the first cut in the process of identifying the most dangerous candidates. Subsequent analyses of the pathogens would then assess the nature of any barriers that limit the establishment of pathogens in human populations (e.g., the absence of suitable arthropod vectors for large proportions of the year).

### Durability

Although durability in various external environments was quantified in detail by microbiologists during the first half of this century (49), modern studies have paid this attribute little attention. Evolutionary considerations, however, indicate that it should be one of the first variables quantified when a new pathogen is being studied. If a new, directly transmitted pathogen can remain viable in the external environment for many days to many weeks, it falls in the category of especially dangerous pathogens. If, for example, Ebola virus were viable upon natural desiccation for weeks instead of hours, its level of host exploitation and potential for transmission from exploited hosts would not be so mismatched, and it, like smallpox virus, would pose a much more serious threat. Durability in the external environment depends largely on environmental conditions (49), and thus assessments of viability should cover all feasible environmental conditions.

### Vector-borne Transmission

The most serious threat involved in vector-borne transmission comes from pathogens that can be maintained by human/mosquito cycles but are absent from suitable areas because of historical accidents or past eradication campaigns. Dengue and malaria are members of this category; they have the potential to spiral out of control immediately upon release into areas with suitable vectors. Nonevolutionary analyses of emerging infections recognize the threat posed by these pathogens because their damaging effects on human populations are known.

Vector-borne pathogens that have not used humans as the primary vertebrate host but may be capable of doing so represent less easily recognized threats. Evolutionary considerations heighten concern because such vector-borne pathogens are expected to become increasingly harmful as they become adapted to human/vector cycles of transmission (16).

Rift Valley fever virus provides an example. For most of this century, this virus was believed to infect humans only as dead-end hosts. Although it was vector-borne in ungulates, humans were seen as acquiring the infection either when involved in the slaughtering process or when bitten by mosquitoes that had acquired infection from other vertebrates. Recent outbreaks have spread to an extent consistent with substantial human/mosquito cycling, but the existence of such cycling has not been conclusively documented. If human/mosquito cycling is occurring, the door is open for further adaptation to humans and for evolution of increased virulence in humans, increased efficiency of human/vector transmission, and increased spread through human populations. Rift Valley fever virus viremias seem sufficient for human/mosquito cycling, and the lethality of the largest outbreaks was particularly high, as one would expect if some evolution toward increased virulence accompanied a temporary establishment of human/mosquito cycles (50-51). To assess the long-term threat posed by Rift Valley fever virus and to block this virus should it prove to be particularly threatening, we need to emphasize the following research priorities: 1) study the transmission of Rift Valley fever virus in human/mosquito cycles, 2) assess the potential for such transmission over extended periods, and 3) evaluate the effects of such transmission on virus virulence.

All emerging vector-borne pathogens need not be viewed as equally threatening. For example, *Borrelia burgdorferi*, the agent of Lyme disease (an emerging vector-borne pathogen in human populations in North America), does not need to be monitored to avoid its establishment as a human pathogen because once emerged, it does not threaten to spiral out of control; it is tick-borne, and ongoing human/tick cycles are not feasible because of the limited exposure of infected humans to susceptible tick populations of the appropriate instar. Tick- and mite-borne rickettsiae do not present a great threat for similar reasons.

### Sexual Transmission

The tradeoff concerning sexually transmitted pathogens may prove particularly useful in identifying pathogens that are capable of sexual transmission and have cell tropisms that would cause severe damage if host exploitation increased but have not had high potential for sexual transmission. Human T-cell lymphotropic virus (HTLV) is in this category, even though by nonevolutionary criteria it could be dismissed because it has been geographically widespread in humans for a long time (1). HTLV type 1 (HTLV-I) is less damaging than HIV; it kills or severely handicaps 5% to 10% of the people it infects, generally decades after infection. Although HTLV-I and HIV infections share many characteristics, HTLV does not have HIV's high mutation rate and hence does not have the potential for staying ahead of immune responses and eventually decimating the immune system. Instead, HTLV relies on modes of transmission that do not expose it to the immune system: proviral replication through stimulation of host cell proliferation and transmission through cell-to-cell contact. A concern with HTLV is that a high potential for sexual transmission may favor increased rates of viral replication leading to increased exposure to the immune system and increased mutation rates (48).

A preliminary step toward evaluating the threat posed by the emergence of particularly virulent HTLVs is assessing whether HTLVs exposed to different levels of potential for sexual transmission vary in virulence. HTLV-I infections tend to lead to leukemias and lymphomas at younger ages in Jamaica, where the potential for sexual transmission is high, than in Japan, where potential for sexual transmission is low (48). This difference also occurs among North Americans of Japanese and Caribbean descent (52), who presumably are infected predominantly (if not exclusively) by Japanese and Caribbean HTLVs, respectively. The inherent virulence and mutation-proneness of the Japanese and Caribbean HTLVs need to be assessed. Similarly, HTLV virulence needs to be better studied in regions of Africa where it has been long endemic to determine whether variations in HTLV virulence are correlated with the potential for sexual transmission.

Although mutation-prone sexually transmitted viruses that infect critical cell types are particularly threatening, sexually transmitted viruses in general deserve special attention. Even if a sexually transmitted virus invades only epithelial cells and replicates with low mutation rates, a high potential for sexual transmission may lead to evolution of increased lethality. Death caused by HTLV-induced lymphomas and leukemias is one manifestation of the danger posed by an RNA virus that replicates substantially in its DNA form and hence is in a middle area within the spectrum of mutation-proneness. HPVs illustrate dangers posed by sexually transmitted viruses that, because they are DNA viruses, are even further away from HIV on the mutation-proneness continuum. The mechanism by which HPV nudges infectious cells toward cancer is associated with increased viral replication; moreover, high potential for sexual transmission (as indicated by the number of lifetime sex partners) is a strong risk factor for infection with the more oncogenic HPV serotypes but not for the mild HPV serotypes (53). This association supports the idea that reductions in the potential for sexual transmission should cause evolution of reduced HPV virulence. Specifically, as the potential for sexual transmission decreases, the risk for acquiring the oncogenic serotypes (vs benign serotypes) should disproportionately decrease. Similarly, if interventions prevent the potential for sexual transmission from increasing, the emergence of oncogenic HPV serotypes should be disproportionately suppressed.

### Waterborne Transmission

Although such pathogens as *Vibrio cholerae* O139 and *Shigella dysenteriae* type 1 threaten emergence in countries with inadequate water supplies, the threat is much lower in countries with safe water supplies. Although such pathogens continue to be brought into the countries with safe water supplies by travelers and commerce, the pathogens show little potential for emergence. For example, a major epidemic of *S. dysenteriae* type 1 spread from Guatemala through Central America during the early 1970s. It entered the United States in several places but dissipated without any great effort at containment. Its transmission was studied in a Los Angeles neighborhood, where each infection gave rise on average to about 0.4 new infections (54). Without amplification by waterborne transmission, this outbreak, like other introductions in the United States, was self-limited (54). The situation at the other end of Central America was similar. The *S. dysenteriae* epidemic dissipated as it moved into Costa Rica, where water supplies were relatively pure (L. J. Mata, pers. comm.).

### Attendant-borne Transmission

Emerging hospital-acquired pathogens may pose one of the greatest and most controllable threats to people in countries like the United States, where more than 5% of hospital admissions and about 14% of intensive care patients acquire infections during their stay (55-57). According to some estimates, nosocomial infections rank among the ten leading causes of death in the United States (56), with dangerous bloodstream infections approximately doubling during the 1980s (58).

Although high virulence has been documented in pathogens involved in nosocomial outbreaks (59-63), the damage caused by nosocomial pathogens has generally been attributed to the state of hospitalized patients, who may be compromised by underlying disease, immunosuppressive drugs, and invasive procedures. These factors, however, do not explain why nosocomial pathogens, such as *Staphylococcus aureus* often cause symptomatic infections in hospital staff (60) but rarely in persons in the outside community. They also do not explain the association between the extent of nosocomial transmission and the virulence of infection, or the differences in symptomatic infections among otherwise healthy babies (17,20,41). In a New York City hospital, for example, where attendant-borne transmission rates were very low, only approximately one of 30 babies with *S. aureus* were symptomatic (64). Among nosocomial outbreaks of endemic disease, the analogous proportion may be 5- to 10-fold higher (65).

Without an evolutionary framework for understanding pathogen virulence, researchers would have no reason for expecting to find particularly virulent endemic pathogens in hospitals. The only serious attempts to explain the apparently high-level of pathogen virulence in hospitals involved the linking of virulence to another characteristic associated with hospitals: antibiotic resistance. The emergence of antibiotic-resistant organisms in hospitals in concert with the use of the antibiotics (66) led researchers to conclude that high levels of antibiotic use caused the emergence of resistant organisms and to speculate that antibiotic-resistant organisms might be inherently more virulent than their antibiotic-sensitive counterparts (67). Yet when infections caused by resistant nosocomial organisms are compared with sensitive (generally nosocomial) infections, the former are only sometimes found to be associated with more severe infections. Even when they are associated with more severe disease (62,63), any differences in inherent virulence tend to be confounded with other factors, such as increased severity due to lowered effectiveness of antibiotics. The increased severity of disease, however, is sometimes associated with resistance to antibiotics other than the one being used (61), suggesting that the increased damage is not simply a result of ineffective antibiotics. The presence of virulence-enhancing bacterial characteristics in damaging, resistant nosocomial strains (63,68) also suggests a link between nosocomial transmission, antibiotic resistance, and virulence: antibiotic-resistant strains may have been particularly virulent because they were nosocomial, but this virulence was not apparent in many of the comparisons because the sensitive strains were also nosocomial.

Although the controversy regarding virulence and antibiotic resistance in hospital-acquired infections can be explained by the hypothesized connection between attendant-borne transmission and the evolution of both virulence and antibiotic resistance, none of the investigations of the topic made measurements that would allow assessment of the connection between attendant-borne transmission and the emergence of variants with increased virulence. The critical measure is the harmfulness per person housing the organisms in question, and the critical comparison is between nosocomial and community-acquired strains. Among persons that harbor nosocomial strains of *S. aureus*, for example, the proportion that show symptomatic infection could be compared with the analogous proportion of matched persons who are harboring community strains. After virulence-enhancing mechanisms are well understood, pathogens can be assayed for their virulence directly. Thus *Clostridium difficile* pathogens isolated from prolonged nosocomial outbreaks are predicted to be more toxigenic than *C. difficile* isolated from the outside community. Similarly, nosocomial *Escherichia coli* are predicted to have virulence-enhancing characteristics (e.g., invasiveness, adherence) (69) more often than community strains.

Further knowledge about virulence enhancing mechanisms and development of techniques for rapid detection (e.g., [72-75]) should offer opportunities for carefully controlled experiments to test whether reduction in attendant-borne transmission causes a greater decline in the inherent virulence of nosocomial pathogens in experimental hospitals than in control hospitals in which interventions are not imposed. Long-term follow-up should clarify the degree to which attendant-borne transmission may foster the emergence of virulent variants among both established human pathogens (e.g., *S. aureus*, *E. coli*) and new or newly recognized pathogens (e.g., *Serratia* spp., and *Pseudomonas aeruginosa*).

Harmful, often antibiotic-resistant, hospital-acquired pathogens can readily emerge beyond a hospital's boundary, when patients are moved, or attendants move between hospitals; the documentation is particularly strong for dangerous variants of *E. coli* and *S. aureus* (62,74-78). The degree to which emerging nosocomial pathogens spill over to generate outbreaks in the outside community is not well understood, but evidence suggests that this spillover represents a substantial threat when the organisms can infect healthy people. When large-scale communitywide epidemics of pathogenic *E. coli* have occurred, for example, transmission in hospitals often was strongly implicated. During 1953 and 1954, an *E. coli* epidemic advanced up the East Coast of the United States from the Carolinas through New England; "As it spread, explosive outbreaks were limited to institutions, hospital wards, and newborn nurseries" (59). A focal study of the U.S. Army Hospital at Fort Belvoir, Virginia, indicated that the epidemic strain was brought into the hospital by infected people in the community, with the proportion of inpatient to outpatient cases reversing dramatically during the hospital's 5-month outbreak (59). Similarly, during the winter of 1961, in an outbreak in Chicago and adjacent communities in Indiana, about 5% of the infants were affected, and nearly half of the affected infants had direct or indirect contact with one of the 29 involved hospitals just before their illnesses (75).

Studies of *S. aureus* have also shown that nosocomial and community outbreaks are sometimes synchronous with transmission occurring in both directions between the hospital and the outside community (79-80). The long-term consequences of emergence of nosocomial strains for the outside community, however, still need to be assessed. The possibility that nosocomial pathogens may tend to be not only more resistant to antibiotics, but also more inherently virulent lends some urgency to this need.

Almost no work has been done to determine the potential of pathogens thought to be almost exclusively associated with nosocomial infection (e.g., *Enterococcus*, *C. difficile*) to take hold in the outside community. The high durability in the external environment of many nosocomial pathogens heightens the need for additional information. Durable pathogens that can infect uncompromised hosts (e.g., antibiotic-resistant *S. aureus* and to a lesser extent *C. difficile*) possess the basic characteristics that damaging organisms need to spread in the outside community. Durable organisms unable to infect healthy people pose a relatively low threat, but this inability is often presumed. Any transmission of durable nosocomial organisms like *P. aeruginosa* from patients after discharge heightens the threat to the outside community by providing an avenue for further adaptation to humans. Molecular analyses that allow reconstruction of epidemiologic patterns (e.g., molecular phylogenetics) could be used to improve assessments of the degree to which nosocomial pathogens can emerge in the outside community; such studies need to provide quantitative assessments not only of the threats posed by nosocomial pathogens in their current state, but also of their potential to breach by evolution the barriers that have inhibited their broader spread in the past.

### Conceptual Innovation, Explanatory Power, and Precision

### Dangerous Emergences of the Past

Each of the organisms that caused devastating epidemics over the past 5 centuries, would have been identified as an extremely dangerous pathogen by the criteria proposed here. *Y. pestis*, for example, is durable in the external environment (49) and is vector-borne. Its threat is lower now than centuries ago when fleas and rats were abundant domiciliary inhabitants, but it still represents a threat where these hosts are present.

The periodic emergence of yellow fever in European and American cities during the 18th and 19th centuries took a heavy toll; the 1878 epidemic, for example, killed about a quarter of the population of Memphis, Tennessee (81). If yellow fever virus were first encountered today, it would be recognized as an important threat because it is vector-borne and can be transmitted indefinitely through human/mosquito cycles.

With regard to the emergence of virulent variants from established pathogens, the influenza viruses circulating at the Western Front during World War I would be considered dangerous because barriers to transmission from immobile hosts were removed by cultural practices and because influenza virus is mutation prone (17,20). It is, therefore, not surprising that the Western Front has been identified as the source of the highly lethal variants of the 1918 influenza pandemic and that a pandemic of this severity has never recurred (17). More importantly, evolutionary considerations suggest that such a lethal pandemic will not recur unless influenza viruses are again exposed to opportunities that allow transmission from immobile hosts, as they are on poultry farms where highly lethal influenza outbreaks periodically emerge (17).

### Uncertainty about the Dangerous Epidemics of the Future

These arguments about the evolution of virulence provide only coarse approximations of the selective processes in pathogen populations. To determine whether the implications of these arguments need to be substantially modified, we need empirical studies that evaluate these arguments against alternative explanations. Considering the current state of uncertainty, some might argue that it is dangerous to incorporate the current coarse understanding of the evolution of virulence into policy making. But failing to incorporate this understanding is dangerous.

If we do not adjust investments to take into account the evolutionary arguments, and the arguments prove correct, the reduction in death and illness per unit investment will be lower than it could have been. If we do adjust investments on the basis of these evolutionary arguments, and the arguments prove wrong, the nonevolutionary benefits of the investments would still be obtained.

Although the precise mechanisms that increase virulence in pathogens in the high-risk categories still need to be clarified, the associations (Table 1) are strong. One could argue, for example, that durable or waterborne pathogens are more harmful because hosts tend to pick up a greater diversity of genotypes from the environment when pathogens are more durable or are mixed in water; if the within-host genetic variability of such pathogens is greater, they would have more potential for within-host competition, which could favor the evolution of increased virulence. By this argument, factors such as durability, vector-borne transmission, and waterborne transmission would increase virulence indirectly by increasing within-host genetic variation. With regard to the prevention of the emergence of highly virulent disease, uncertainties about mechanisms are not critical. Whether the effects of these factors are direct or indirect, elimination of the factors should discourage the emergence of severe disease and favor the decrease of highly virulent pathogens.

Decisions to invest in interventions without certainty about mechanisms is not new to the health sciences. The hygienic interventions to control hospital acquired diseases and the purification of water supplies to control cholera were appropriately advocated on the basis of epidemiologic data (from Ignaz Semmelweis and John Snow) a half century before the causative agents of these or any other infectious diseases were first identified. Jenner's smallpox vaccine program was accepted globally more than a century before viruses were discovered or the mechanisms by which vaccines provide protection were understood. Even now the mechanisms by which the immune system provides protection encompass major areas of uncertainty. This uncertainty is evidenced, for example, by the controversies about the importance of the different legs of the immune system (such as cytotoxic T cells, neutralizing antibody, and subsets of helper T cells) in HIV pathogenesis.

If the evolutionary arguments are correct, the emergence of the most harmful diseases can be countered not only for pathogens that are recognized as threats but also for those posing threats that are not yet recognized. Providing pure water supplies, reducing attendant-borne transmission, and reducing vector-borne transmission preferentially from ill people (e.g., by providing mosquito-proof houses [17]) should guard against the emergence of virulent pathogens, whether the pathogens are unidentified or are highly virulent variants of identified human pathogens. An understanding of the evolutionary determinants of virulence may thus make surveillance and prompt intervention much more manageable.

The emphasis thus is on suppression of the emergence of particularly virulent variants rather than suppression of the emergence of new disease organisms. The expectation is that the frequency of disease will drop even though the frequency of individuals harboring organisms may decline little if at all. The data on decentralization of nursery/maternity wards, for example, indicate that the rates of nosocomial infection decline among mothers and babies, even though the rates at which babies harbor pathogens (colonization plus infection) do not decline (82). Indeed the disagreement about the value of rooming-in as a mode of infection control (82) can be attributed to a failure to distinguish the prevalence of disease organisms from the prevalence of disease. Controversies about the value of waterborne transmission can be traced to a similar failure (17).

The lead article of the first issue of this journal was entitled, "Emerging infections: getting ahead of the curve" (4). I propose that integrating evolutionary principles with epidemiology would enhance our ability to stay ahead of the curve. Evolutionary insights should increase our ability to distinguish emerging pathogens according to the long-term threat that they pose and thereby adjust investments in accordance with the threat. Knowledge of the evolution of virulence should also guide us to identify for each pathogen the critical data that will allow us to make this assessment. Finally, evolutionary considerations should allow identification of infrastructural investments that will guard against the most dangerous pathogens, even if they are not blocked by surveillance and containment efforts and even if they have not yet been identified or are never identified as emerging pathogens.

*Dr. Ewald is a professor in the Department of Biology at Amherst College. Trained in ecology and evolutionary biology, he works at the interface of these areas with epidemiology, focusing on the evolution of virulence among infectious diseases of humans and insects.*

### References

1. Morse SM. Emerging viruses: defining the rules for viral traffic. Perspect Biol Med 1991;34:387-409.
2. Morse SM. Examining the origins of emerging viruses, In: Morse SM, editor. Emerging viruses. New York: Oxford, 1993;10-28.
3. Morse SM. Factors in the emergence of infectious disease. Emerging Infectious Diseases 1995;1:7-15.
4. Satcher D. Emerging infections: getting ahead of the curve. Emerging Infectious Diseases 1995; 1:1-6.
5. Henderson DA. Surveillance systems and intergovernmental cooperation. In: Morse SM, editor. Emerging viruses. New York: Oxford, 1993;283-9.
6. Holland J. Replication error, quasispecies populations, and extreme evolution rates of RNA viruses. In: Morse SM, editor. Emerging viruses. New York, Oxford, 1993;203-18.
7. Krause RM. Foreword. In: Morse SM, editor. Emerging viruses. New York: Oxford, 1993;xvii-xix.
8. McNeill WH. Patterns of disease emergence in history. In: Morse SM, editor. Emerging viruses. New York: Oxford, 1993;29-36.
9. Smith T. Parasitism and disease. Princeton: Princeton University Press, 1934.
10. Dubos R. Man adapting. New Haven, Conn: Yale University Press, 1965.
11. Burnet FM, White DO. Natural history of infectious disease, 4th ed. Cambridge: Cambridge University Press, 1972.
12. Thomas L. Notes of a biology-watcher: Germs. N Engl J Med 1972;247:553-5.
13. Levin S, Pimentel D. Selection of intermediate rates of increase in parasite-host systems. American Naturalist 1981;117:308-15.
14. Levin BR, Allison AC, Bremermann HJ, Cavalli-Sforza LL, Clarke BC, B\Frentzel-Beyme R, et al. Evolution of parasites and hosts. Group report, In: Anderson RM, May RM, editors. Population Biology of Infectious Diseases. Berlin: Springer-Verlag 1982;213-43.
15. Anderson RM, May RM. Coevolution of hosts and parasites. Parasitology 1982;85:411-26.
16. Ewald PW. Host-parasite relations, vectors, and the evolution of disease severity. Annual Review of Ecology and Systematics 1983;14:465-85.
17. Ewald PW. Evolution of Infectious Disease. New York: Oxford University Press, 1994.
18. Morens DM, Marchette NJ, Chu MC, Halstead SB. Growth of dengue type-2 virus isolates in human peripheral blood leukocytes correlates with severe and mild dengue disease. Am J Trop Med Hyg 1991;45:644-51.
19. Gulig PA, Doyle TJ. The *Salmonella typhimurium* virulence plasmid increases the growth rate of salmonellae in mice. Infect Immun 1993;61:504-11.
20. Ewald PW. Transmission modes and the evolution of virulence, with special reference to cholera, influenza and AIDS. Human Nature 1991;2:1-30.
21. Åsjö B, Morfeldt-Månson L, Albert J, Biberfeld G, Karlsson A, Lidman K, et al. Replicative capacity of human immunodeficiency virus from patients with varying severity of HIV infection. Lancet 1986;334:660-2.
22. ChengMayer C, Seto D, Tateno M, Levy JA. Biologic features of HIV-1 that correlate with virulence in the host. Science 1988;240:80-2.
23. Albert J, Böttiger B, Biberfeld G, Fenyö EM. Replicative and cytopathic characteristics of HIV-2 and severity of infection. Lancet 1989;333:852-3.
24. Fenyö EM, Albert J, Åsjö B. Replicative capacity, cytopathic effects and cell tropism of HIV. AIDS 1989;3:S5-12.
25. Levy JA. Human immunodeficiency viruses and the pathogenesis of AIDS. JAMA 1989;261:2997-3006.
26. Tersmette M, Gruters RA, de Wolf F, de Goede REY, Lange JMA, Schellekens PTA, et al. Evidence for a role of virulent human immunodeficiency virus (HIV) variants in the pathogenesis of acquired immunodeficiency syndrome: Studies on sequential HIV isolates. J Virol 1989;63:2118-25.
27. Tersmette M, Lange JMA, de Goede REY, de Wolf F, Eefink Shaattenkerk JKM, Schellekens PTA, et al. Association beteween biological properties of human immunodeficiency virus variants and risk for AIDS and AIDS mortality. Lancet 1989;333:983-5.
28. Ma X, Sakai K, Sinangil F, Golub E, Volsky DJ. Interaction of a noncytopathic human immunodeficiency virus type 1 (HIV-1) with target cells: Efficient virus entry followed by delayed expression of its RNA and protein. Virology 1990;176:184-94.
29. Schneweis K E, Kleim J-P, Bailly E, Niese D, Wagner N, Brackmann HH. Graded cytopathogenicity of the human immunodeficiency virus (HIV) in the course of HIV infection. Med Microbiol Immunol 1990;179:193-203.
30. Gruters RA, Terpstra FG, DeGoede REY, Mulder JW, DeWolf F, Schellekens PTA, et al. Immunological and virological markers in individuals progressing from seroconversion to AIDS. AIDS 1991;5:837-44.

31. Schellekens PTA, Tersmette M, Roos MTL, Keet RP, Dewolf F, Coutinho RA, et al. Biphasic rate of CD4+ cell count decline during progression to AIDS correlates with HIV-1 phenotype. AIDS 1992;6:665-9.
32. Hirsch I, Salaun D, Brichacek B, Chermann JR. HIV-1 cytopathogenicity—genetic difference between direct cytotoxic and fusogenic effect. Virology 1992;186:647-54.
33. Koot M, Keet IPM, Vos AHV, DeGoede REY, Roos MTL, Coutinho, RA, et al. Prognostic value of HIV-1 syncytium-inducing phenotype for rate of CD4+ cell depletion and progression to AIDS. Ann Intern Med 1993;118:681-8.
34. Connor RI, Mohri H, Cao YZ, Ho DD. Increased viral burden and cytopathicity correlate temporally with CD4+ T lymphocyte decline and clinical progression in human immunodeficiency virus type 1- infected individuals. J Virol 1993;67:1772-7.
35. Connor R I, Ho DD. Human immunodeficiency virus type 1 variants with increased replicative capacity develop during the asymptomatic stage before disease progression. J Virol 1994;68:4400-8.
36. Fisher SG. Epidemiology: a tool for the study of human papillomavirus-related carcinogenesis. Intervirology 1994;37:215-25.
37. Puel J, Lheritier D, Guyader M, Izopet J, Briant L, Tricoire J, et al. Viral load and mother-to-infant HIV transmission. Lancet 1992;340:859.
38. Weiser B, Nachman S, Tropper P, Viscosi KH, Grimson R, Baxter G, et al. Quantitation of human immunodeficiency virus type 1 during pregnancy: relationship of viral titer to mother-to-child transmission and stability of viral load. Proc Natl Acad Sci USA 1994;91:8037-41.
39. Kaye JN, Cason J, Pakarian F, Jewers R, Kell B, Bible J, et al. Viral load as a determinant for transmission of human papillomavirus type 16 from mother to child. J Med Virol 1994;44:415-21.
40. Adjorloto-Johnson G, De Cock KM, Ekpini E, Vetter KM, Sibailly T, Brattegaard K, et al. Prospective comparison of mother-to-child transmission of HIV-1 and HIV-2 in Abidjan, Ivory Coast. JAMA 1994;272:462-6.
41. Ewald PW. Cultural vectors, virulence, and the emergence of evolutionary epidemiology. Oxford Surveys in Evolutionary Biology 1988;5:215-45.
42. Ewald PW. Waterborne transmission and the evolution of virulence among gastrointestinal bacteria. Epidemiol Infect 1991;106:83-119.
43. Weniger BG, Tansuphaswadikul S, Young NL, Pau CP, Lohsomboon P, Yindeeyoungyeon W, et al. Differences in immune function among patients infected with distinct Thailand HIV-1 strains, In: Proceedings of the Tenth International Conference on AIDS. Yokohama, Japan, 7-12 August 1994; Abstract O12C.
44. Kitayaporn D, Tansuphaswadikul S, Lohsomboon P, Pannachet K, Kaewkungwal J, Limpakarnjanarat K, et al. Survival of AIDS patients in the emerging epidemic in Bangkok, Thailand. J Acquir Immune Defic Syndr Hum Retrovirol 1996;11:77-82.
45. Learmont J, Tindall B, Evans L, Cunningham A, Cunningham P, Wells J, et al. Long-term symptomless HIV-1 infection in recipients of blood products from a single donor. Lancet 1992;340:863-7.
46. Cao YZ, Qin LM, Zhang LQ, Safrit J, Ho DD. Virologic and immunologic characterization of long-term survivors of human immunodeficiency virus type 1 infection. N Engl J Med 1995;332:201-8.
47. Kirchhoff F, Greenough TC, Brettler DB, Sullivan JL, Desrosiers RC. Brief report: absence of intact nef sequences in a long-term survivor with nonprogressive HIV-1 infection. N Engl J Med 1995;332:228-32.
48. Ewald PW. Evolution of mutation rate and virulence among human retroviruses. Phil Trans Roy Soc Lond, Ser B Biol Sci 1995;346:333-43.
49. Mitscherlich E, Marth EH. Microbial Survival in the Environment. Berlin, Germany: Springer-Verlag, 1984.
50. Meegan JM. Rift Valley fever in Egypt: an overview of the epizootics in 1977 and 1978. Contributions to Epidemiology and Biostatistics 1981;3:100-13.
51. House JA, Turell MJ, Mebus CA. Rift valley fever: present status and risk to the western hemisphere. Ann NY Acad Sci 1992;653:233-42.
52. Levine PH, Manns A, Jaffe ES, Colclough G, Cavallaro A, Reddy G, et al. The effect of ethnic differences on the pattern of HTLV-I-associated T-cell leukemia/lymphoma (HATL) in the United States. Int J Cancer 1994;56:177-81.
53. Franco EF, Villa LL, Ruiz A, Costa MC. Transmission of cervical human papillomavirus infection by sexual activity: differences between low and high oncogenic risk types. J Infect Dis 1995;172:756-63.
54. Weissman JB, Murton KI, Lewis JN, Friedemann CHT, Gangarosa EJ. Impact in the U.S. of the Shiga dysentery pandemic of Central America and Mexico: A review of surveillance data through 1972. J Infect Dis 1974;129:218-23.
55. Dixon RE. Effect of infections on hospital care. Ann Intern Med 1978;89:749-53.
56. Haley RW, Culver DH, White JW, Morgan WM, Emori TG. The nationwide nosocomial infection rate: A new need for vital statistics. Am J Epidemiol 1985;121:159-67.
57. Raju TNK, Kobler C. Improving handwashing habits in the newborn nurseries. Am J Med Sci 1991;302:355-8.
58. Pittet D, Wenzel RP. Nosocomial bloodstream infections. Arch Intern Med 1995;155:1177-84.
59. Belnap D, O'Donnell JJ. Epidemic gastroenteritis due to *Escherichia coli* 0:111. J Pediat 1955;47:178-93.
60. Rountree P, Freeman BM. Infections caused by a particular phage type of *Staphylococcus aureus*. Med J Austr 1955;2:157-61.
61. Jessen O, Rosendal K, Bülow P, Faber V, Eriksen KR. Changing staphylococci and staphylococcal infections. A ten-year study of bacteria and cases of bacteremia. N Engl J Med 1969;281:627-35.

62. Locksley RM, Cohen ML, Quinn TC, Tompkins LS, Coyle MB, Kirihara JM, et al. Multiply antibiotic-resistant *Staphylococcus aureus*: introduction, transmission, and evolution of nosocomial infection. Ann Intern Med 1982;97:317-24.
63. Rello J, Torres A, Ricart M, Valles J, Gonzalez J, Artiga A, et al. Ventilator-associated pneumonia by *Staphylococcus aureus*. Am J Respir Crit Care Med 1994;150:1545-9.
64. Holzman R, Florman A, Lyman M. Gentamicin resistant and sensitive strains of *S. aureus*. Factors affecting colonization and virulence for infants in a special care nursery. Am J Epidemiol 1980;112:352-61.
65. Gezon HM, Rogers KD, Thompson DJ, Hatch TF. Some controversial aspects in the epidemiology of hospital nursery staphylococcal infections. Am J Public Health 1960;50:473-84.
66. Gezon HM, Schaberg MJ, Klein JO. Concurrent epidemics of *Staphylococcus aureus* and group A *streptococcus* disease in a newborn nursery-control with penicillin G and hexachlorophene bathing. Pediatrics 1973;51:383-90.
67. Craven DE, Reed C, Kollisch N, DeMaria A, Lichtenberg D, Shen K, et al. A large outbreak of infections caused by a strain of *Staphylococcus aureus* resistant to oxacillin and aminoglycosides. Am J Med 1981;71:53-8.
68. Huebner J, Pier GB, Maslow JN, Muller E, Shiro H, Parent M, et al. Endemic nosocomial transmission of *Staphylococcus epidermidis* bacteremia isolates in a neonatal intensive care unit over 10 years. J Infect Dis 1994;169:526-31.
69. Donnenberg MS, Kaper JB. Enteropathogenic *Escherichia coli*. Infect Immun 1992;60:3953-61.
70. Donnenberg MS, Tacket CO, James SP, Losonsky G, Nataro JP, Wasserman SS, et al. Role of the eaeA gene in experimental enteropathogenic *Escherichia coli* infection. J Clin Invest 1993;92:1412-7.
71. Franke J, Franke S, Schmidt H, Schwarzkopf A, Wieler LH, Baljer G, et al. Nucleotide sequence analysis of enteropathogenic *Escherichia coli* (EPEC) adherence factor probe and development of PCR for rapid detection of EPEC harboring virulence plasmids. J Clin Microbiol 1994;32:2460-3.
72. Schmidt H, Plaschke B, Franke S, Russman H, Schwarzkopf A, Heesemann J, et al. Differentiation in virulence patterns of *Escherichia coli* possessing eae genes. Med Microbiol Immunol 1994;183:23-31.
73. Spitz J, Yuhan R, Koutsouris A, Blatt C, Alverdy J, Gecht G. Enteropathogenic *Escherichia coli* adherence to intestinal epithelial monolayers diminishes barrier function. Am J Physiol 1995;268:G374-9.
74. Rogers KB, Koegler SJ. Inter-hospital cross-infection of epidemic infantile gastro-enteritis associated with type strains of *Bacterium coli*. Journal of Hygiene 1951;49:152-61.
75. Marcy SM: Microorganisms responsible for neonatal diarrhea, in Remington JS, Klein JO, editors. Infectious diseases of the fetus and newborn infant. Philadelphia: WB Saunders, 1976:892-978.
76. Saroglou G, Cromer M, Bisno AL. Methicillin-resistant *Staphylococcus aureus*:interstate spread of nosocomial infections with emergence of gentamicin-methicillin resistant strains. Infection Control 1980;1:81-9.
77. Pavillard R, Harvey K, Douglas D, Hewstone A, Andrew J, Collopy B, et al. Epidemic of hospital-acquired infection due to methicillin-resistant *Staphylococcus aureus* in major Victorian hospitals. Med J Aust 1982;1:451-4.
78. Lyon BR, Iuorio JL, May JW, Skurray RA. Molecular epidemiology of multiresistant *Staphylococcus aureus* in Australian hospitals. J Med Entomol 1984;17:79-89.
79. Gooch JJ, Britt EM. *Staphylococcus aureus* colonization and infection in newborn nursery patients. American Journal of Diseases of Children 1978;132:893-6.
80. Saravolatz LD, Markowitz N, Arking L, Pohlod D, Fisher E. Methicillin-resistant *Staphylococcus aureus*. Ann Intern Med 1982;96:11-6.
81. Keating JM. History of the yellow fever epidemic of 1878 in Memphis, Tennessee. Cincinnati, Ohio: Wrightson, 1879.
82. Daschner F. Infectious hazards in rooming-in systems. J Perinat Med 1984;12:3-6.
